# Two cases of hemolymphangioma in the thoracic spinal canal and spinal epidural space on MRI: The first report in the literature

**DOI:** 10.1097/MD.0000000000009524

**Published:** 2017-12-29

**Authors:** Xingchen Pan, Yutong Dong, Tingting Yuan, Yuzhu Yan, Dan Tong

**Affiliations:** a Department of Radiology; b Department of Gastroenterology, The First Hospital of Jilin University, Changchun, Jilin Province, China.

**Keywords:** hemolymphangioma, meningioma, MRI, schwannoma, spinal

## Abstract

**Rationale:**

Hemolymphangioma is a rare, noninvasive benign tumor of mesenchymal origin resulting from malformation of vascular and lymphatic vessels. The incidence of hemolymphangioma in the spinal canal is low.

**Patient concerns:**

This report describes 2 patients with a lesion located in the thoracic spinal canal or spinal epidural space, who were misdiagnosed with suspected meningioma or suspected schwannoma, respectively, based on magnetic resonance imaging (MRI).

**Diagnoses:**

Hemolymphangioma.

**Interventions:**

The application of a surgery was designed to treat the 2 patients.

**Outcomes:**

2 patients stated that symptoms were improved after the operation.

**Lessons:**

This report should raise awareness among clinicians that careful image analysis and consideration of patient history and pathology is required for accurate differential diagnosis of hemolymphangioma in the spinal canal and spinal epidural space.

## 1 Introduction

Hemolymphangioma is a rare, noninvasive benign tumor of mesenchymal origin resulting from malformation of vascular and lymphatic vessels. Hemolymphangioma can be congenital or acquired, but are most frequently congenita,^[1]^ occurring at an estimated incidence of 1.2 to 2.8 per 1000 newborn infants.^[2]^ Previously, reports have described hemolymphangioma in the abdomen, mediastinum, extremities, oral region, and on the tongue and orbit.^[3]^ To the author's knowledge, there are no reports of hemolymphangiomas in the spinal canalin international papers. This article presents 2 cases of hemolymphangioma in the spine that were misdiagnosed based on findings from magnetic resonance imaging (MRI)

## 2 Case studies

Approval was obtained from the Ethics Committee of The First Hospital of Jilin University, and all subjects gave their informed consent to participate.

**Figure 1:**
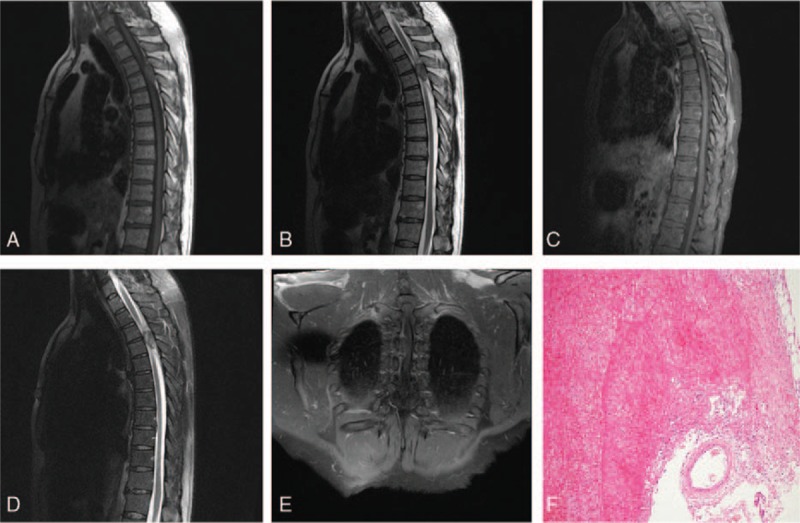
Case 1: A–C Sagittal magnetic resonance imaging (MRI) showing a lesion located in the spinal canal. The lesion appears hyperintense on T1-weighted images (A); the lesion appears hypointense and hyperintense on T2-weighted and fat suppression images (B, C); D, E: Contrast enhanced sagittal and coronal MRI showing an uneven, slightly enhanced shadow; F: pathology showing a thrombus (H&E ×10).

Case 1: A 58-year-old male presented to our hospital with thoracic back pain, with weakness and hypoesthesia in both lower extremities. The patient was administered rehydration therapy for 1 day, and his thoracic back pain was slightly alleviated, however, weakness with hypoesthesia in the lower extremities, gradually worsened. Physical examination, including the Glasgow Coma Scale, revealed that the patient was conscious (spontaneous eye response: +4) verbally fluent (oriented: +5) but had no motor response (+1). The patient had normal upper limb strength (grade 5/5), but grade 0/5 (no contraction) lower limb strength, although muscle tension was normal. The patient was experiencing numbness from T6 to T12 and had pain in the mid-thoracic spine (T4 to T6). Residual neurological examination revealed no obvious abnormalities. The MRI scan demonstrated a 2.5 cm × 1.5 cm lesion at T3/T4 that appeared hyperintense on T1-weighted images (Fig. 1A), and hypointense and hyperintense on T2-weighted images (Fig. 1B) and fat suppression images (Fig. 1C), as well as compression of the spinal cord. Contrast enhanced the lesion, showing an uneven, slightly enhanced shadow (Fig. 1D and E). The patient was diagnosed with a suspected meningioma. Following surgical resection of the suspected meningioma, pathology revealed a brown irregular 1.0 cm × 1.2 cm × 2.7 cm mass with a rough surface covered in blood clots. The pathological diagnosis was hemolymphangioma and thrombosis (Fig. 1F).

**Figure 2:**
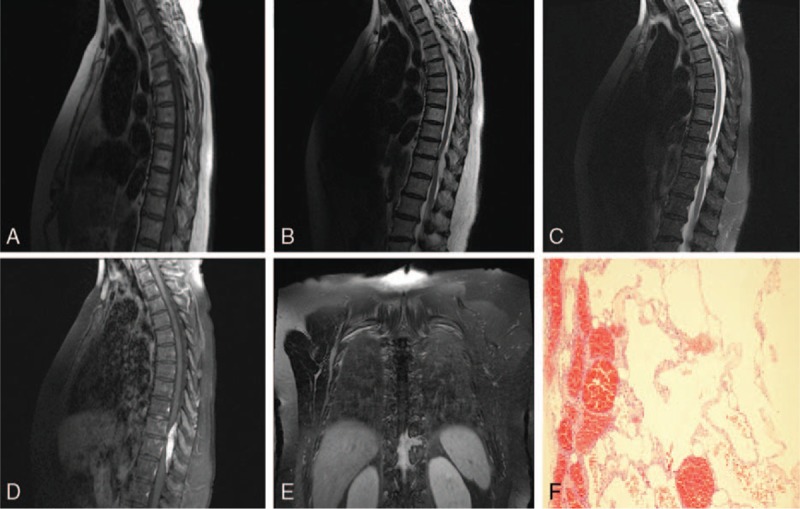
Case 2: A–C: Sagittal magnetic resonance imaging (MRI) showing a lesion located in the spinal canal. The lesion appears hypointense on T1-weighted images (A); the lesion appears hyperintense on T2-weighted and fat suppression images (B, C); D, E: Contrast enhanced the lesion showed irregular, severe enhanced shadow in the spinal epidural space at T10 to T12 and intervertebral perforation at T10/11. F: Pathology showing a hemangioma on the left and a lymphangioma on the right (H&E ×10).

Case 2: A 60-year-old female presented to our hospital with hypoesthesia of the left thigh. She had been suffering from symptoms for 4 years and had difficulty in walking for 2 years. Physical examination, including the Glasgow Coma Scale, revealed that the patient was conscious (spontaneous eye response: +4) verbally fluent (oriented: +5,) but had no motor response (+1). The patient had normal upper limb strength (grade 5/5) and grade 4/5 lower limb strength. Sensitivity to pain, temperature, and coarse touch of both lower limbs were decreased, and there was a positive bilateral Babinski sign. The MRI scan revealed a 6.1 cm × 0.9 cm lesion in the spinal epidural space at T10 to T12 that infringed upon the adjacent intervertebral foramen and appeared hypointense on T1-weighted images (Fig. 2A) and hyperintense on T2-weighted (Fig. 2B) and fat suppression images (Fig. 2C). Contrast enhanced the lesion showing an irregular, severely enhanced shadow in the spinal epidural space at T10 to T12 and intervertebral perforation at T10/11 (Fig. 2D and E). The patient was diagnosed with a suspected schwannoma. Following surgical resection of the suspected schwannoma, pathology showed a solid reddish-brown irregular 0.5 cm × 1.8 cm × 5.0 cm mass. The pathological diagnosis was hemolymphangioma (Fig. 2F).

## 3 Discussion

Hemolymphangiomas present as cystic or cavernous lesions that consist of dilated veins and lymphatics interspersed with normal stromal tissue and vasculature; dilated vessels may contain thrombosis.^[4]^ On immunohistochemistry analysis, hemolymphangiomas are CD31 and D2-40 positive.^[5]^ Hemolymphangiomas may be congenital or acquired. Congenital hemolymphangiomas result from an obstruction of the venolymphatic communication between the systemic circulation and the dysembryoplastic vascular tissue.^[3]^ Acquired hemolymphangiomas occur due to inadequate lymph drainage and damage to the lymphatics resulting from surgery or trauma. Evidence suggests that imaging characteristics of hemolymphangiomas vary according to the location in the body, proportion of blood and lymphatic vasculature, and imaging modality. Ultrasound, computed tomography, and MRI are useful for the diagnosis of hemolymphangiomas, with findings dependent on the amount of water-based substance and number of blood vessels in the lesion.^[6]^ The patients in this report underwent contrast-enhanced MRI. In Case 1, MRI findings indicated a lesion resembling a meningioma, a commonintra-spinal canal tumor, and a more likely diagnosis than spinal hemolymphangioma. However, careful analysis of imaging results should distinguish between meningioma and hemolymphangioma, as meningiomas arise from the arachnoid layer of the meninges, most have a dural tail attaching them to the dura mater, and some contain calcified deposits.^[7]^ MRI imaging of Case 1 revealed hyperintense signals in T1- and T2-weighted images of the lesion. Combined with pathological results, these findings were indicative of subacute thrombosis, which is considered a characteristic feature of hemolymphangioma; this suggests that MRI is an effective tool for the diagnosis of this lesion. Hemolymphangioma was considered responsible for the patient's back pain and weakness in the lower extremities as it is a slow growing lesion that is associated with gradual onset of clinical symptoms. The acute onset of serious dysfunction of the lower limbs was attributed to thrombosis.

The patient in Case 2 had lower limb symptoms for 4 years. This is consistent with chronic progressive spinal cord compression and the presence of a slow growing mass. MRI imaging of Case 2 revealed a lesion in the spinal epidural space that was infringing upon the adjacent intervertebral foramen. This resulted in a diagnosis of an atypical schwannoma, the most common extramedullary spinal tumor. Schwannomas generally appear as rounded lesions on imaging; however, they often protrude out of neural exit foramen and forma dumbbell shape. Schwannomas are hypointense on T1-weighted images and hyperintense on T2-weighted images with necrotic and cystic areas.^[8]^ As hemolymphangiomas show “crawling” progressive growth, rather than the expansion growth characteristic of schwannomas,^[9]^ careful analysis of imaging results should distinguish between the 2.

Both patients in this study were treated with surgical resection, which is considered the most effective management for hemolymphangioma. Recurrence rates are reported at 10% to 27% following complete removal of a hemolymphangioma.^[3]^

In conclusion, the incidence of hemolymphangioma in the spine is low, and clinical diagnosis remains challenging. This report describes 2 patients with hemolymphangioma in the thoracic spinal canal or spinal epidural space who were misdiagnosed with a suspected meningioma or suspected schwannoma, respectively. Our findings suggest that MRI combined with patient history and pathology is required for an accurate diagnosis of hemolymphangiomas in the spinal canal and spinal epidural space. This report should raise awareness among clinicians of the importance of MRI, patient history, and pathology in the differential diagnosis of hemolymphangioma.
